# Parental attitudes and adolescent empathy in a rural sociocultural context in Türkiye

**DOI:** 10.3389/fpsyg.2026.1944408

**Published:** 2026-10-22

**Authors:** Selma Baraç, Talat Sarikavak

**Affiliations:** 1 Karşıyaka Girls’ Vocational and Technical Anatolian High School, Central District, Bingöl, Türkiye; 2 Department of Psychiatry, Faculty of Medicine, Atlas University, Istanbul, Türkiye; 3 Acibadem Fulya Hospital, Istanbul, Türkiye

**Keywords:** adolescent mental health, authoritarian parenting, cross-cultural psychiatry, empathy, parenting styles, rural population, Türkiye

## Abstract

**Background:**

Adolescence is a critical period for the consolidation of identity and socio-emotional skills such as empathy, and parenting attitudes represent influential contextual factors. Most evidence on this relationship derives from Western samples, and it remains unclear whether the associations reported there hold in traditional family systems. This study examined the relationship between perceived parental attitudes and empathy among adolescents in a rural sociocultural context in Türkiye.

**Methods:**

In this cross-sectional study, 458 high school students aged 14–18 in Bingöl completed the Toronto Empathy Questionnaire (TEQ), the Parental Attitude Scale (PAS) and a sociodemographic form. Data were collected in two single-sex schools authorized by the provincial education directorate. Analyses comprised *t-*tests, one-way ANOVA with effect sizes, Pearson correlations and hierarchical multiple regression. The Benjamini–Hochberg procedure was applied to control the false discovery rate across group comparisons.

**Results:**

Authoritarian parenting was negatively associated with empathy both bivariately (*r* = −0.162) and after adjustment for demographic characteristics and the remaining parenting dimensions (*β* = −0.170, *p* = 0.002). Protective/demanding parenting showed a small positive association that emerged more clearly under adjustment (*β* = 0.131, *p* = 0.006), whereas democratic parenting was not associated with empathy (*β* = −0.026, *p* = 0.648). A smaller number of siblings was associated with higher empathy. The model explained 12.5% of the variance (adjusted *R*^2^ = 0.113). Notably, the democratic and authoritarian subscales were themselves moderately positively correlated (*r* = 0.586).

**Conclusion:**

In this context, the controlling dimension of parenting carried the association with empathy while the warmth dimension did not, and the two dimensions were not opposed to one another. These findings support attention to authoritarian control in family-based approaches to adolescent socio-emotional development, and indicate that the cross-cultural applicability of classical parenting typologies warrants direct examination rather than assumption.

## Introduction

Adolescence represents a transitional period characterized not only by biological and cognitive maturation but also by profound psychosocial changes (Ericson, 1950; Branje, 2022). It is widely regarded as the stage in which identity formation becomes central, with social identity development being particularly salient (Branje, 2022; Crocetti et al., 2023). In this process, adolescents negotiate increasing autonomy from parents, often resulting in conflicts around independence, values, and decision-making (Crocetti et al., 2016; Buehler, 2020; Garcia et al., 2021). Parent-adolescent conflict intensity tends to increase from early to middle adolescence, especially as adolescents push for greater self-direction and parents adjust to renegotiated roles in the family (Scully et al., 2020; Mastrotheodoros et al., 2024). Such conflicts, while stressful, provide opportunities for adolescents to practice negotiation, emotional regulation, and perspective-taking (Carlo et al., 2011; Goering and Mrug, 2021; Yadav et al., 2021). At the same time, peer relationships gain increasing prominence, offering contexts for intimacy, mutual validation, and the rehearsal of complex social roles (Boele et al., 2019; Zhou et al., 2023). Together, these dynamics shape the adolescent’s emerging social identity and communication competencies, which prepare the individual for active participation in adult social life (Scully et al., 2020; Zhou et al., 2023; Mastrotheodoros et al., 2024).

Within these processes, empathy plays a crucial role. Empathy develops gradually, beginning with affective responsiveness in early childhood and progressing toward more complex cognitive perspective-taking during adolescence (Gaspar and Esteves, 2022). By this stage, adolescents acquire the ability to integrate emotional understanding with abstract reasoning, allowing them to grasp not only how others feel but also why they feel that way (van Lissa et al., 2014; Gaspar and Esteves, 2022). Recent longitudinal evidence supports that there are heterogeneous trajectories in empathy development: some adolescents show moderate stable levels of empathic concern, while others demonstrate increasing perspective-taking over time (van Lissa et al., 2014). Notably, higher perspective-taking tends to predict increases in empathic concern rather than vice versa.

This developmental expansion strengthens skills such as emotional regulation, conflict management, and prosocial behaviors (Farrant et al., 2012; Yadav et al., 2021). Adolescence, when identity and social roles are being consolidated, is a period in which empathy becomes a cornerstone of socio-emotional development: it enables adolescents to build deeper friendships, establish mutual trust, and engage in cooperative group dynamics (Balconi and Canavesio, 2016; Farrell and Vaillancourt, 2021, 2023). Identity development theory in psychology and sociology highlights that through feedback from parents and peers, adolescents form social identities that guide their attitudes and behaviors in social settings (e.g., peer groups, schools) (Ekman et al., 1987). Empathic capacities refined during adolescence contribute to the crystallization of long-term social patterns and moral frameworks (van Lissa et al., 2014; Gaspar and Esteves, 2022). These capacities also play a role in adolescents’ readiness for civic engagement and intergroup relations, preparing them for active participation in their communities.

Parental attitudes are among the most decisive contextual factors shaping adolescent personality and socio-emotional development (Carlo et al., 2011; Farrant et al., 2012; Pinquart, 2017; Bruysters and Pilkington, 2023; Jabbar et al., 2024). In classical typologies, parenting styles are often grouped as democratic (authoritative), authoritarian, permissive, neglectful, or protective (Kuppens and Ceulemans, 2019; Lansford et al., 2021). Adolescents who perceive their parents as democratic tend to report higher emotional understanding, prosocial orientation, and stronger peer relationships (van Lissa et al., 2014). Recent cross-cultural research further supports this finding, indicating that authoritative parenting fosters empathy, self-regulation, and social competence in diverse adolescent populations (Carlo et al., 2011; Pinquart, 2017). In contrast, authoritarian parenting—characterized by rigid control and limited responsiveness—has been associated with poorer socio-emotional outcomes, on the grounds that such approaches may foster compliance while undermining autonomy and socio-emotional flexibility (Masud et al., 2019; Avci and Sak, 2021; Wang et al., 2021). Evidence concerning protective and over-involved parenting is less consistent. Some accounts emphasize that overprotection restricts opportunities for adolescents to exercise perspective-taking and independent social functioning (Bruysters and Pilkington, 2023) whereas others note that protective attitudes also convey attentiveness and involvement, which may support empathic responsiveness (Farrant et al., 2012; Masud et al., 2019; Wang et al., 2021). This tension remains unresolved, particularly in collectivistic and traditional family contexts, where protectiveness may carry different social meaning than in Western samples (Lansford et al., 2021). These findings highlight that, while various parental approaches coexist, democratic parenting has been proposed to play a uniquely facilitative role in supporting empathy during adolescence (Özmen and Atli, 2021).

Taken together, existing evidence highlights that adolescence is a sensitive period for the consolidation of social identity and socio-emotional skills, with empathy serving as a central capacity for personal adjustment, family functioning, and societal cohesion. Parenting attitudes—particularly democratic, authoritarian, and protective-demanding styles—emerge as critical contextual factors that may either foster or hinder the development of empathy. Given that democratic parenting is typically less prevalent in traditional family systems, examining whether its association with empathy holds in such a context constitutes a substantive test rather than a replication. However, empirical studies explicitly addressing these associations remain limited within the Türkiye rural cultural context, where family bonds, intergenerational relationships, and parenting practices may differ from Western societies. Considering that adolescence and emerging youth represent formative stages in which both identity and empathy development are consolidated, this period was deliberately chosen for the present study. Accordingly, the study aims to examine how adolescents’ perceptions of democratic, authoritarian, and protective-demanding parental attitudes are associated with their empathy levels, thereby contributing to a more culturally grounded understanding of family influences on socio-emotional development in Türkiye.

## Materials and methods

### Design and setting

This cross-sectional study was conducted in two public secondary schools in the central district of Bingöl, a province in eastern Türkiye, during the spring term of the 2022–2023 academic year.

### Participants and recruitment

Ethical approval was obtained from the Istanbul Gelişim University Ethics Committee (decision no. 2023–04-122, dated 19 April 2023). Institutional authorization was subsequently granted by the provincial Directorate of National Education (Permit no. E-48605746-44-76271589, dated 16 May 2023), which specified the two schools in which data collection was permitted. Both schools are located in the central district; one enrolls female students only and the other male students only. Because each school is single-sex, school and participant sex are fully confounded in the present sample, and any comparison by sex is simultaneously a comparison between the two school contexts. This is addressed in the analytic strategy below and in the Limitations.

All students in grades 9 through 12 attending the two schools on the day of administration were invited to participate (*N* = 495). Inclusion criteria were: age 14–18 years, current enrolment in secondary education, and sufficient literacy to complete self-report questionnaires. Exclusion criteria were a history of psychiatric or neurological disorder, intellectual disability, chronic medical illness, or absence of informed consent.

Of the 495 students invited, 7 were absent on the day of administration, leaving 488 who received questionnaire packets. Fourteen students either declined to participate or did not return their forms, yielding 474 returned questionnaires (97.1% of those approached). Sixteen returned forms were excluded because they showed invariant responding across all items, indicating non-engagement with the task. The final analytic sample therefore comprised 458 adolescents (92.5% of those invited; 93.9% of those who received questionnaires).

Questionnaires were administered in classroom settings during regular school hours by the first author, who is a teacher at one of the participating schools. Students were informed in writing and verbally that participation was voluntary, that responses would be anonymous, that non-participation would carry no academic consequence, and that they could withdraw at any point. Written informed consent was obtained from parents or legal guardians, and written assent was obtained from each student prior to administration. All procedures complied with the 1964 Helsinki Declaration and its subsequent amendments.

### Missing data

No participant had missing values on any study variable with the exception of grade point average (*n* = 1, 0.2%). The single affected case was handled by listwise deletion in the analyses involving that variable (*n* = 457); all other analyses were conducted on the full sample (*N* = 458).

### Assessment tools

Empathy was assessed with the Toronto Empathy Questionnaire (TEQ; Spreng et al., 2009), in its Turkish adaptation (Totan et al., 2012). The Turkish adaptation comprises 13 items rated on a 5-point scale (possible range 13–65), three items of the original 16-item instrument having been removed during adaptation owing to inadequate psychometric performance in Turkish samples. Parental attitudes were assessed with the Parental Attitude Scales (PAS) (Kuzgun and Eldeleklioğlu, 1999), which yields Democratic, Authoritarian and Protective/Demanding subscale scores; internal consistency coefficients reported in the original scale-development study were α = 0.89, 0.82 and 0.78, respectively.

In the present sample, internal consistency was α = 0.748 (ω = 0.756) for the TEQ, and α = 0.733, 0.793 and 0.774 for the Democratic, Authoritarian and Protective/Demanding subscales, respectively, (α = 0.803 for the PAS Total score). All item–rest correlations were positive, confirming correct scoring of reverse-keyed items.

### Statistical analysis

Analyses were conducted in SPSS 24.0 and jamovi 2.7. Normality was evaluated using skewness and kurtosis values, with the interval ±1.5 taken as indicating approximate normality; all scale scores fell within this range.

Two-group comparisons employed independent-samples *t-*tests with Cohen’s d and 95% confidence intervals. Comparisons across three or more categories employed one-way analysis of variance with η^2^ as the effect size and Tukey’s HSD for *post-hoc* contrasts. Bivariate associations were examined with Pearson correlations.

Because Tables 1, 2 comprise a large number of group comparisons, the Benjamini–Hochberg procedure for controlling the false discovery rate was applied at α = 0.05 separately within each outcome family, each family comprising the nine grouping variables tested against a single outcome (*m* = 9). Families were defined by outcome rather than pooled across the tables, on the grounds that a false positive concerning empathy and one concerning a parental attitude dimension address distinct research questions. The procedure retains control of the false discovery rate under positive dependency among tests, which is the expected structure here given that all measures derive from the same respondents. Results that did not survive correction are reported as exploratory.

**Table 1:** Comparison of empathy and parental attitude scores by students’ sociodemographic and academic characteristics.

Characteristic	*n* (%)	Empathy (TEQ)	PAS Democratic	PAS Authoritarian	PAS Protective/ Demanding
Sex					
Female	341 (74.5)	53.03 (7.64)	44.07 (6.17)	26.41 (5.18)	46.27 (7.38)
Male	117 (25.5)	48.89 (7.97)	44.54 (6.36)	26.46 (5.50)	47.94 (8.28)
Test statistic		*t*(456) = 4.99; ***p* < 0.001**†; *d* = 0.54	*t*(456) = −0.72; *p* = 0.475; *d* = −0.08	*t*(456) = −0.08; *p* = 0.936; *d* = −0.01	*t*(456) = −2.04; ***p* = 0.041**; *d* = −0.22
Age (years)					
14	102 (22.3)	52.48 (7.42)	43.20 (5.38)	25.96 (4.87)	46.30 (6.72)
15	94 (20.5)	49.41 (7.63)	45.64 (6.35)	27.24 (4.98)	45.23 (6.90)
16	127 (27.7)	53.24 (7.84)	43.91 (6.41)	26.40 (5.31)	47.48 (8.08)
17	98 (21.4)	52.15 (8.21)	44.93 (5.98)	26.80 (5.97)	47.49 (8.00)
18	37 (8.1)	52.32 (8.60)	42.30 (7.18)	24.70 (4.42)	46.81 (9.06)
Test statistic		*F*(4, 453) = 3.46; ***p* = 0.009**†; *η*^2^ = 0.030	*F*(4, 453) = 3.26; ***p* = 0.012**; *η*^2^ = 0.028	*F*(4, 453) = 1.90; *p* = 0.109; *η*^2^ = 0.017	*F*(4, 453) = 1.53; *p* = 0.192; *η*^2^ = 0.013
Grade level					
9	139 (30.3)	51.35 (7.79)	43.89 (6.00)	26.44 (5.05)	46.37 (6.98)
10	55 (12.0)	49.91 (8.03)	44.31 (6.98)	26.78 (5.57)	45.60 (8.66)
11	195 (42.6)	53.33 (7.77)	44.51 (6.45)	26.78 (5.60)	46.91 (7.70)
12	69 (15.1)	51.07 (8.13)	43.81 (5.37)	25.12 (4.22)	47.67 (7.96)
Test statistic		*F*(3, 454) = 3.81; ***p* = 0.010**†; *η*^2^ = 0.025	*F*(3, 454) = 0.37; *p* = 0.777; *η*^2^ = 0.002	*F*(3, 454) = 1.82; *p* = 0.142; *η*^2^ = 0.012	*F*(3, 454) = 0.88; *p* = 0.451; *η*^2^ = 0.006
Grade point average (*n* = 457)					
85–100	62 (13.6)	52.68 (7.68)	44.27 (6.03)	26.40 (5.48)	47.40 (7.31)
70–84	191 (41.8)	53.41 (7.90)	43.23 (5.87)	25.57 (4.66)	47.57 (7.71)
60–69	107 (23.4)	50.91 (7.16)	44.67 (6.78)	27.44 (5.78)	45.85 (7.55)
50–59	77 (16.8)	49.95 (8.44)	45.68 (6.17)	26.84 (5.54)	45.77 (8.03)
0–49	20 (4.4)	49.70 (8.72)	44.85 (6.12)	27.65 (5.05)	44.50 (6.44)
Test statistic		*F*(4, 452) = 3.96; ***p* = 0.004**†; *η*^2^ = 0.034	*F*(4, 452) = 2.55; ***p* = 0.039**; *η*^2^ = 0.022	*F*(4, 452) = 2.72; ***p* = 0.029**; *η*^2^ = 0.023	*F*(4, 452) = 1.78; *p* = 0.131; *η*^2^ = 0.016

*N* = 458 unless otherwise indicated. Values are M (SD). TEQ, Toronto Empathy Questionnaire; PAS, Parental Attitude Scale. Two-group comparisons used independent-samples *t-*tests with Cohen’s *d*; comparisons across three or more categories used one-way ANOVA with η^2^. Significant Tukey *post-hoc* contrasts: empathy by age, 16 > 15 (*p* = 0.004); empathy by grade level, 11 > 10 (*p* = 0.023); empathy by grade point average, 70–84 > 50–59 (*p* = 0.010); PAS Democratic by grade point average, 50–59 > 70–84 (*p* = 0.029); PAS Authoritarian by grade point average, 60–69 > 70–84 (*p* = 0.024). PAS Total scores are reported in Supplementary Table S1. *p*-values are two-tailed. †Remained significant after Benjamini–Hochberg correction for the false discovery rate, applied separately within each outcome family (*m* = 9 comparisons per outcome, α = 0.05). No comparison involving a PAS subscale survived this correction; those results are therefore reported as exploratory. Bold values indicate comparisons significant at *p* < 0.05 before correction for multiple testing; the dagger symbol indicates results that remained significant after Benjamini-Hochberg correction for the false discovery rate.

**Table 2:** Comparison of empathy and parental attitude scores by family characteristics.

Characteristic	*n* (%)	Empathy (TEQ)	PAS democratic	PAS authoritarian	PAS protective/demanding
Mother’s education					
Illiterate	67 (14.6)	50.20 (7.96)	43.45 (5.86)	26.31 (5.26)	44.78 (8.10)
Primary school	243 (53.1)	52.20 (8.10)	44.32 (6.10)	26.34 (5.28)	46.55 (7.99)
Secondary school	96 (21.0)	52.50 (7.60)	43.91 (6.56)	26.42 (4.98)	47.86 (6.01)
High school	48 (10.5)	52.50 (7.78)	45.10 (6.85)	27.06 (5.74)	48.15 (7.95)
University	4 (0.9)	50.80 (6.18)	44.75 (3.59)	26.25 (6.85)	43.50 (4.12)
Test statistic		*F*(4, 453) = 1.04; *p* = 0.385; *η*^2^ = 0.009	*F*(4, 453) = 0.58; *p* = 0.677; *η*^2^ = 0.005	*F*(4, 453) = 0.20; *p* = 0.939; *η*^2^ = 0.002	*F*(4, 453) = 2.27; *p* = 0.061; *η*^2^ = 0.020
Father’s education					
Illiterate	14 (3.1)	51.43 (8.35)	44.21 (6.20)	25.57 (4.38)	48.00 (8.88)
Primary school	142 (31.0)	51.64 (7.75)	44.56 (6.53)	27.04 (5.41)	46.88 (7.51)
Secondary school	169 (36.9)	52.02 (7.80)	43.81 (5.83)	26.09 (4.94)	46.12 (7.47)
High school	110 (24.0)	51.83 (8.51)	44.52 (6.58)	26.40 (5.65)	47.14 (8.11)
University	23 (5.0)	54.83 (6.98)	43.13 (5.39)	25.78 (5.22)	47.04 (7.10)
Test statistic		*F*(4, 453) = 0.83; *p* = 0.505; *η*^2^ = 0.007	*F*(4, 453) = 0.53; *p* = 0.716; *η*^2^ = 0.005	*F*(4, 453) = 0.84; *p* = 0.501; *η*^2^ = 0.007	*F*(4, 453) = 0.46; *p* = 0.766; *η*^2^ = 0.004
Number of siblings					
1–3	206 (45.0)	52.95 (7.76)	44.81 (6.50)	26.85 (5.54)	47.57 (7.69)
4–5	196 (42.7)	51.70 (7.91)	43.80 (6.24)	26.10 (4.85)	46.50 (7.77)
6 or more	56 (12.2)	49.30 (8.11)	43.34 (4.78)	25.90 (5.54)	44.10 (6.49)
Test statistic		*F*(2, 455) = 4.90; ***p* = 0.008**†; *η*^2^ = 0.021	*F*(2, 455) = 2.00; *p* = 0.137; *η*^2^ = 0.009	*F*(2, 455) = 1.27; *p* = 0.282; *η*^2^ = 0.006	*F*(2, 455) = 4.62; ***p* = 0.010**; *η*^2^ = 0.020
Parents’ living arrangement					
Living together	416 (90.8)	52.00 (7.80)	44.27 (6.21)	26.41 (5.26)	46.69 (7.68)
Living separately	21 (4.6)	50.10 (10.18)	44.52 (6.35)	27.81 (5.23)	48.38 (7.31)
Parent deceased	21 (4.6)	53.60 (8.07)	42.30 (6.26)	25.50 (5.21)	45.20 (7.37)
Test statistic		*F*(2, 455) = 1.02; *p* = 0.363; *η*^2^ = 0.004	*F*(2, 455) = 1.00; *p* = 0.369; *η*^2^ = 0.004	*F*(2, 455) = 1.07; *p* = 0.343; *η*^2^ = 0.005	*F*(2, 455) = 0.89; *p* = 0.411; *η*^2^ = 0.004
Family income					
Income below expenses	158 (34.5)	52.17 (7.99)	44.91 (7.40)	27.35 (5.61)	45.84 (8.04)
Income equal to expenses	228 (49.8)	51.99 (7.89)	43.83 (5.54)	26.19 (5.00)	46.89 (7.80)
Income above expenses	72 (15.7)	51.51 (8.03)	43.76 (5.30)	25.14 (4.98)	48.01 (6.00)
Test statistic		*F*(2, 455) = 0.17; *p* = 0.844; *η*^2^ = 0.001	*F*(2, 455) = 1.62; *p* = 0.199; *η*^2^ = 0.007	*F*(2, 455) = 4.92; ***p* = 0.00**8; *η*^2^ = 0.021	*F*(2, 455) = 2.16; *p* = 0.117; *η*^2^ = 0.009

*N* = 458. Values are M (SD). PAS, Parental Attitude Scale. Comparisons used one-way ANOVA with η^2^. Significant Tukey *post-hoc* contrasts: empathy by number of siblings, 1–3 > 6 or more (*p* = 0.006); PAS Protective/Demanding by number of siblings, 1–3 > 6 or more (*p* = 0.008); PAS Authoritarian by family income, income below expenses > income above expenses (*p* = 0.008). PAS Total scores are reported in Supplementary Table S2. *p*-values are two-tailed. †Remained significant after Benjamini–Hochberg correction for the false discovery rate, applied separately within each outcome family (*m* = 9 comparisons per outcome, α = 0.05). No comparison involving a PAS subscale survived this correction; those results are therefore reported as exploratory. Bold values indicate comparisons significant at *p* < 0.05 before correction for multiple testing; the dagger symbol indicates results that remained significant after Benjamini-Hochberg correction for the false discovery rate.

A hierarchical multiple linear regression was conducted with empathy as the criterion. Sociodemographic variables (sex, age, number of siblings) were entered in Model 1 and all three PAS subscales simultaneously in Model 2. The PAS Total score was not entered because it is the arithmetic sum of the three subscales and is therefore exactly linearly dependent on them. Multicollinearity was assessed using variance inflation factors and tolerance values.

Because the sample comprised only two clusters, and because cluster membership was identical to participant sex, neither multilevel modeling nor cluster-robust standard errors could be estimated. Participant sex was retained in both regression models; since it is collinear with school, its inclusion absorbs between-school variation, and the parental-attitude coefficients may therefore be interpreted as within-school estimates. Residual within-school dependency (for example, shared classroom or teacher effects) could not be modeled and is acknowledged in the Limitations.

All tests were two-tailed and evaluated at α = 0.05.

## Results

### Descriptive statistics

A total of 458 adolescents (74.5% female, *n* = 341; 25.5% male, *n* = 117) participated in the study. The sample size was deemed adequate for the planned statistical analyses, including group comparisons and regression, and the distributions of the continuous variables were suitable for parametric testing. This was confirmed by skewness and kurtosis values falling within the acceptable range of ±1.5 for all scale scores.

The mean empathy score was 51.97 (SD = 7.93), while mean scores for the parental attitude dimensions were as follows: 44.19 (SD = 6.21) for the democratic subscale, 26.42 (SD = 5.26) for the authoritarian subscale, and 46.70 (SD = 7.64) for the protective/demanding subscale. The total PAS score was 117.32 (SD = 14.52).

An examination of parental education revealed that mothers had notably lower educational attainment compared to fathers. PAS Total scores by sociodemographic and family characteristics are presented in Supplementary Tables S1, S2.

### Comparison of subgroups

Group comparisons were conducted using independent samples t-tests and one-way ANOVAs to assess how empathy and parental attitude scores varied across sociodemographic and family-related variables (see Tables 1, 2).

A significant gender difference was found in both empathy and the protective/demanding parental attitude subscale. Female students reported higher empathy levels than male students (*p* < 0.001). In contrast, male students perceived significantly higher levels of protective/demanding parenting (*p* = 0.041).

Academic achievement was related to empathy, with higher-achieving students reporting greater empathic capacity (*p* = 0.004). Students in the 70–84 band perceived lower democratic parenting than those in the 50–59 band (*p* = 0.029) and lower authoritarian parenting than those in the 60–69 band (*p* = 0.024); neither contrast survived correction for multiple testing.

Among family-related variables, parental education was unrelated to empathy and to parental attitude scores. Number of siblings was negatively associated with empathy (*p* = 0.008) and with protective/demanding parenting (*p* = 0.010); it was unrelated to democratic parenting (*p* = 0.137).

Family income was associated with perceived authoritarian parenting: adolescents from households reporting income below expenses perceived higher authoritarian parenting than those from households with income above expenses (*p* = 0.008). This contrast did not survive correction for multiple testing.

### Correlation analysis

Pearson correlation coefficients among age, number of siblings, empathy and the parental attitude dimensions are presented in Table 3.

**Table 3:** Bivariate correlations among age, number of siblings, empathy, and parental attitude dimensions (*N* = 458).

Variable	1	2	3	4	5	6
1. Age	–					
2. Number of siblings	−0.002	–				
3. Empathy (TEQ)	0.040	−0.126**	–			
4. PAS democratic	−0.000	−0.063	−0.079	–		
5. PAS authoritarian	−0.024	−0.044	−0.162***	0.586***	–	
6. PAS protective/demanding	0.070	−0.121*	0.092*	0.367***	0.160***	–
7. PAS Total	0.028	−0.107*	−0.044	0.834***	0.698***	0.742***

*N* = 458; df = 456 for all coefficients. TEQ, Toronto Empathy Questionnaire; PAS, Parental Attitude Scale. Inter-correlations among the PAS subscales are reported in full. The PAS Total score is the arithmetic sum of the three subscales and is therefore linearly dependent on them; it is reported here for completeness but was not entered into the regression models. *p < 0.05, **p < 0.01, ***p < 0.001.

Number of siblings was negatively correlated with empathy (*r* = −0.126, *p* = 0.007). Age was not significantly associated with empathy at the bivariate level (*r* = 0.040, *p* = 0.393). Authoritarian parenting was negatively correlated with empathy (*r* = −0.162, *p* < 0.001), whereas Protective/Demanding parenting showed a weak positive correlation that only marginally reached significance (*r* = 0.092, *p* = 0.050). Democratic parenting was not significantly correlated with empathy (*r* = −0.079, *p* = 0.093).

Among the PAS subscales, the strongest association was between the Democratic and Authoritarian dimensions (*r* = 0.586, *p* < 0.001), followed by Democratic–Protective/Demanding (*r* = 0.367, *p* < 0.001); the Authoritarian–Protective/Demanding association was comparatively weak (*r* = 0.160, *p* < 0.001). The substantial positive association between the Democratic and Authoritarian dimensions is inconsistent with the orthogonal structure assumed in classical parenting typologies and is considered further in the Discussion. The PAS Total score correlated strongly with all three subscales (*r* = 0.698–0.834), as expected given that it is their arithmetic sum.

### Regression analysis

Results of the hierarchical regression are presented in Table 4. In Model 1, sex, age and number of siblings were entered as sociodemographic predictors. This model was significant, *F* (3, 454) = 13.60, *p* < 0.001, *R*^2^ = 0.082, adjusted *R*^2^ = 0.076.

**Table 4:** Hierarchical multiple regression predicting adolescent empathy (*N* = 458).

(A) Overall model tests								
Model	*R*	*R* ^2^	Adj. *R*^2^	Δ*R*^2^	*F*	df1	df2	*p*
1	0.287	0.082	0.076	–	13.60	3	454	<0.001
2	0.353	0.125	0.113	0.042	10.70	6	451	<0.001
Model 1 vs. 2				0.042	7.23	3	451	<0.001

(B) Regression coefficients							
Predictor	*B*	*SE*	95% CI [LL, UL]	*t*	*p*	β	VIF
Model 1							
Intercept	40.512	4.926	[30.833, 50.192]	8.22	<0.001		
Sex	4.801	0.847	[3.136, 6.466]	5.67	<0.001	0.264	1.08
Age	0.684	0.295	[0.103, 1.265]	2.31	0.021	0.108	1.07
Number of siblings	−0.749	0.238	[−1.216, −0.282]	−3.15	0.002	−0.142	1.00
Model 2							
Intercept	43.305	5.649	[32.204, 54.406]	7.67	<0.001		
Sex	4.942	0.833	[3.306, 6.578]	5.94	< 0.001	0.272	1.08
Age	0.613	0.290	[0.042, 1.183]	2.11	0.035	0.097	1.08
Number of siblings	−0.716	0.235	[−1.177, −0.255]	−3.05	0.002	−0.136	1.02
PAS authoritarian	−0.256	0.082	[−0.418, −0.095]	−3.11	0.002	−0.170	1.53
PAS protective/demanding	0.136	0.050	[0.038, 0.234]	2.74	0.006	0.131	1.19
PAS democratic	−0.034	0.074	[−0.179, 0.111]	−0.46	0.648	−0.026	1.73

N, 458. PAS, Parental Attitude Scale; CI, confidence interval; LL, lower limit; UL, upper limit; B, unstandardized regression coefficient; β, standardized regression coefficient; VIF, variance inflation factor. Sex was coded 1 = female, 0 = male. Model 1 includes the sociodemographic covariates; Model 2 additionally includes all three PAS subscales entered simultaneously. All tolerance values exceeded 0.58 and all VIF values were below 2, indicating no multicollinearity among the predictors. The PAS Total score was not entered into the models because it is the arithmetic sum of the three subscales and is therefore exactly linearly dependent on them. *p-*values are two-tailed.

In Model 2, all three PAS subscales were entered simultaneously. The addition significantly improved model fit, Δ*R*^2^ = 0.042, *F* (3, 451) = 7.23, *p* < 0.001; the full model was significant, *F* (6, 451) = 10.70, *p* < 0.001, *R*^2^ = 0.125, adjusted *R*^2^ = 0.113. Authoritarian parenting emerged as a significant negative predictor of empathy (B = −0.256, SE = 0.082, 95% CI [−0.418, −0.095], *β* = −0.170, *p* = 0.002) and Protective/Demanding parenting as a significant positive predictor (B = 0.136, SE = 0.050, 95% CI [0.038, 0.234], *β* = 0.131, *p* = 0.006), whereas Democratic parenting was not a significant predictor (B = −0.034, SE = 0.074, 95% CI [−0.179, 0.111], *β* = −0.026, *p* = 0.648). Confidence intervals are reported on the unstandardised scale. Sex and number of siblings remained significant, and age reached significance in the adjusted models.

Multicollinearity diagnostics indicated no problematic collinearity among the predictors: the highest variance inflation factor was 1.73 (Democratic subscale), and all tolerance values exceeded 0.58. The PAS Total score was not entered into the model because it is the arithmetic sum of the three subscales (44.19 + 26.42 + 46.70 = 117.32) and is therefore exactly linearly dependent on them.

Two apparent discrepancies between the bivariate and multivariate results warrant clarification. First, age was unrelated to empathy at the bivariate level (*r* = 0.040) but reached significance in the regression models (*β* = 0.097). This reflects suppression: age was associated with participant sex in this sample, and the age–empathy association emerged once sex was held constant. Second, the association between Protective/Demanding parenting and empathy was stronger in the adjusted model (*β* = 0.131) than at the bivariate level (*r* = 0.092), reflecting the same mechanism. Both coefficients should therefore be interpreted as conditional rather than marginal associations. The group comparison across age bands (Table 1) similarly reflects a non-monotonic distribution—the only significant pairwise contrast was between 15- and 16-year-olds—rather than a linear developmental trend.

## Discussion

The descriptive findings of this study reflect the characteristics of a predominantly rural and traditional sociocultural context. Differences between mothers’ and fathers’ education levels, the relatively high number of siblings, and the predominance of lower income levels all support this profile. Such indicators are consistent with the demographic structure commonly observed in traditional family systems, where socioeconomic limitations and gender disparities in education remain salient (Cindoglu et al., 2011; Erdoğan, 2016; Kara and Selcuk, 2023). Importantly, these contextual features were also echoed in the parental attitude scores: overall averages suggested a tendency toward more controlling and protective approaches, rather than consistently democratic parenting styles (Lansford et al., 2021). Notably, the Democratic and Authoritarian subscales were themselves moderately positively correlated in this sample, suggesting that warmth and control may not function as opposing dimensions in this context—a pattern we return to below. This pattern underscores the influence of broader sociocultural conditions on family dynamics and highlights the necessity of interpreting adolescents’ empathy development within the framework of traditional familial and community structures (Erdoğan, 2016; Cindoglu et al., 2011).

Gender and age differences observed in the present study are in line with developmental theories and empirical evidence on adolescence (Gaspar and Esteves, 2022). Female students scored higher on empathy, with a moderate effect size [*d* = 0.54, 95% CI (0.32, 0.75)]. This is consistent with a large body of research using self-report measures of empathy, typically attributed to gendered socialization processes and greater emotional expressiveness (Farrant et al., 2012; Gaspar and Esteves, 2022). The present design, however, does not permit this difference to be attributed to sex specifically: data were collected in two single-sex schools, so sex and school context are fully confounded, and the observed difference reflects their combined contribution.

Age was not associated with empathy at the bivariate level (*r* = 0.040, *p* = 0.393), and group differences across age bands were small (η^2^ = 0.030) and non-monotonic, the only significant contrast lying between 15- and 16-year-olds. The association reached significance only once sex was held constant (*β* = 0.097, *p* = 0.035), reflecting suppression—age and sex were themselves correlated (*r* = −0.257). We do not interpret this coefficient as evidence of a developmental trajectory.

Academic achievement was associated with empathy, with higher-performing students reporting greater empathic capacity. The directionality of this association cannot be established within a cross-sectional design. Three interpretations remain equally compatible with the data: that empathic capacity supports academic engagement, that academic success affords social experiences conducive to perspective-taking, or that a third factor—such as executive functioning or verbal ability—underlies both (van Lissa et al., 2014; Balconi and Canavesio, 2016; Goodhew and Edwards, 2022). A further caution applies here: because the two participating schools differ in academic program, grade point average is not measured on a common scale across the sample, and part of this association may reflect between-school differences in grading norms rather than individual variation in achievement.

When perceptions of parental attitudes were examined in relation to adolescents’ characteristics, the patterns were weaker than those observed for empathy. Male students reported higher levels of protective/demanding parenting than female students, whereas the two groups did not differ on the authoritarian dimension. Neither comparison survived correction for multiple testing, and—as noted above—sex is confounded with school context in this sample. We therefore do not interpret these differences substantively.

Perceptions of democratic parenting also varied across age bands (η^2^ = 0.028), but not in a monotonic fashion: the significant contrasts were between 15-year-olds and both the youngest and the oldest groups, and this comparison did not survive correction. The previously suggested interpretation—that recognition of parental behavior as democratic declines as adolescents pursue greater autonomy—is not supported by the present pattern (Boele et al., 2019; Özmen and Atli, 2021; Domingo, 2025). Academic achievement was related to perceptions of parental attitudes, with students in the 50–59 band reporting higher democratic parenting than those in the 70–84 band (*p* = 0.029), and students in the 60–69 band reporting higher authoritarian parenting than the 70–84 band (*p* = 0.024). These contrasts were likewise exploratory, as neither survived correction. Any interpretation—for instance, that academically successful adolescents experience less parental involvement of either kind because parents perceive them as more self-sufficient (Zahed Zahedani et al., 2016; De Sanctis and Mesurado, 2022)—must therefore be treated as a hypothesis for future testing rather than a finding of this study.

Among family variables, parental education was unrelated to both empathy and parental attitude scores, with all effect sizes negligible (η^2^ ≤ 0.020). This is consistent with research suggesting that parental warmth and interaction quality matter more for empathy development than formal educational attainment (Farrant et al., 2012; Özmen and Atli, 2021; Wang et al., 2021; Domingo, 2025). Family income was associated with perceived authoritarian parenting rather than with protective/demanding parenting: adolescents from households reporting income below expenses perceived higher authoritarian parenting than those from households with income above expenses. This is consistent with the family stress model, in which economic pressure increases parental irritability and reliance on coercive control (Morris et al., 2021; Kara and Selcuk, 2023). This contrast was exploratory and did not survive correction, though its direction is theoretically coherent.

Number of siblings was negatively associated with empathy (η^2^ = 0.021; 1–3 siblings > 6 or more, *p* = 0.006), and this was the only family-level comparison to survive correction for multiple testing. It was also associated with lower protective/demanding scores (*p* = 0.008), which did not survive correction. The effect was small, and the design permits no inference about mechanism. The dilution account—that larger households afford less individualized parental attention (Jambon et al., 2019; McVey Neufeld et al., 2024)—is plausible but untested here; it is equally consistent with the data that family size and parenting orientation are jointly determined by cultural and economic conditions that themselves bear on socio-emotional development, in which case family size would be a marker rather than a cause (Lansford et al., 2021; Morris et al., 2021).

The clearest finding of this study concerns authoritarian parenting. Adolescents who perceived their parents as more rigid and controlling reported lower empathy, both at the bivariate level and after adjustment for sex, age, number of siblings and the remaining parental attitude dimensions. The association was therefore robust to the inclusion of competing predictors, and its magnitude falls within the range typically reported in the parenting literature, where associations between parenting dimensions and adolescent socio-emotional outcomes commonly fall between *r* = 0.15 and *r* = 0.25 (Pinquart, 2017; Masud et al., 2019).

The association held after the Democratic subscale was entered into the same model, indicating that it does not simply reflect the absence of warmth but is attributable to the controlling dimension itself. Internal consistency of the Authoritarian subscale was adequate (α = 0.793), so attenuation through measurement error is unlikely.

Interpretatively, rigid control may constrain the interactional conditions under which perspective-taking is practiced. Where parental directives are issued without explanation and disagreement is discouraged, adolescents have fewer occasions to articulate their own position, encounter a differing one, and negotiate between them (Goering and Mrug, 2021; Wang et al., 2021). This account remains inferential: the design cannot distinguish it from the reverse pathway or from a shared antecedent.

Protective/demanding parenting showed a positive association with empathy. At the bivariate level this was weak and of borderline significance (*r* = 0.092, *p* = 0.050); in the adjusted model the coefficient was larger and more clearly distinguishable from zero (B = 0.136, SE = 0.050, 95% CI [0.038, 0.234], *β* = 0.131, *p* = 0.006). The strengthening under adjustment reflects suppression: protective/demanding scores were higher in the male-only school, which also reported lower empathy, so the two associations partially mask one another until sex is held constant. The adjusted coefficient is therefore a conditional rather than a marginal association.

The magnitude nonetheless remains modest—approximately 1.5% of unique variance—and we do not claim that protective parenting fosters empathy. What the finding indicates is that protectiveness and authoritarian control are not interchangeable forms of involvement in this context: entered simultaneously, they carried coefficients of opposite sign. Protective attentiveness may convey engagement even where it restricts autonomy (Carlo et al., 2011; Farrant et al., 2012) whereas rigid control appears to carry no such compensating component.

Democratic parenting was not associated with empathy at either level of analysis and group comparisons involving this subscale did not survive correction for multiple testing. Because this dimension is central to the study’s framework, the null warrants scrutiny. It appears informative rather than indeterminate: the 95% confidence interval for the standardised coefficient excludes effects larger than |β| = 0.14, the study was powered at 0.80 to detect |β| ≥ 0.16, and the subscale’s internal consistency was adequate (α = 0.733), making attenuation through measurement error an unlikely explanation. An association of the magnitude typically reported in Western samples would therefore have been detectable. The result thus contrasts with evidence linking authoritative parenting to greater emotional understanding and prosocial orientation (Carlo et al., 2011; Pinquart, 2017).

A structural feature of the data bears on this null. The Democratic and Authoritarian subscales were moderately positively correlated (*r* = 0.586)—the strongest inter-subscale association in the sample, and far stronger than that between the Authoritarian and Protective/Demanding dimensions (*r* = 0.160). Classical typologies, which treat warmth-with-autonomy and control-without-responsiveness as opposing configurations, do not predict this (Kuppens and Ceulemans, 2019).

Common-method variance and the structure of the instrument may contribute, since both scales were completed by the same adolescent and the subscales may index the intensity of parental engagement more than its style. A substantive reading is also available. In traditional and collectivistic family systems, warmth and control may not be experienced as antagonistic: Kağıtçıbaşı’s model of the autonomous-related self describes configurations in which emotional interdependence persists alongside directive parenting (Kagitcibasi, 2005), and cross-cultural work has repeatedly found that the Baumrind typology does not separate cleanly outside Western samples (Lansford et al., 2021).

On this reading the null concerns the applicability of the construct rather than the absence of an effect: if the democratic dimension does not stand in opposition to the authoritarian one here, it is not to be expected to predict empathy as it does elsewhere. What the data show is that the controlling component carried the association with empathy while the warmth component did not—a dissociation that measures distinguishing behavioral from psychological control would be well placed to examine.

Considered as a whole, the model accounted for 12.5% of the variance in empathy (adjusted *R*^2^ = 0.113), with the parental attitude dimensions contributing 4.2% beyond the sociodemographic variables (*p* < 0.001). This increment is the substantive result: perceived parenting carries information about adolescent empathy that demographic position does not. The more informative result lies in which dimension carried that increment. Of the three subscales entered simultaneously, the controlling dimension predicted empathy and the warmth dimension did not, while protective involvement predicted in the opposite direction from control. This dissociation is what the simultaneous specification makes visible and what a composite parenting score would have obscured. It suggests that the relevant distinction here is not between warm and controlling parenting—dimensions that were themselves positively correlated—but between forms of control that permit negotiation and forms that do not.

The practical implication is correspondingly narrow. The consistent finding across analyses concerns authoritarian control, not family structure. Where interventions are considered, parenting programs that reduce reliance on rigid control and support autonomy-granting practices are the components most directly aligned with the present findings (Bruysters and Pilkington, 2023; Cunha et al., 2023).

### Limitations

Several limitations qualify these findings. First, the cross-sectional design precludes causal or developmental inference. Associations reported here are equally consistent with parenting shaping empathy, with adolescents’ empathic tendencies influencing how they perceive and elicit parental behavior, and with shared antecedents affecting both. Second, both parental attitudes and empathy were assessed through adolescent self-report. This introduces common-method variance, which may inflate associations among measures, and captures perceived rather than enacted parenting. The moderate positive correlation between the Democratic and Authoritarian subscales may partly reflect this. Multi-informant designs incorporating parental report and observational measures would address both concerns. Third, data were collected in two schools authorized by the provincial education directorate, one enrolling female students only and the other male students only. Sex and school context are therefore fully confounded, and the sex difference in empathy reflects their combined contribution. Because grading norms differ between academic programs, grade point average is likewise not measured on a common scale across the sample, and the association between achievement and empathy should be read with this in mind. With only two clusters, neither multilevel modeling nor cluster-robust standard errors could be estimated; retaining sex in the regression models absorbs between-school variation, so that the parenting coefficients are within-school estimates, but residual dependency arising from shared classroom or teacher effects could not be modeled. Fourth, although the return rate was high (97.1% of students approached), participation was voluntary and questionnaires were administered by a teacher at one of the participating schools, which may have exerted an implicit influence on the decision to take part; 16 forms were excluded for invariant responding, so the least engaged respondents are under-represented. Fifth, the Turkish adaptation of the TEQ yields a single total score and does not distinguish affective from cognitive empathy, and the PAS does not separate behavioral from psychological control—distinctions that bear directly on the interpretations offered above. Sixth, effect sizes were small throughout, and comparisons involving the parental attitude subscales did not survive correction for multiple testing. Those results are reported as exploratory and require replication in adequately powered samples. Finally, the sample was drawn from a single rural province with a traditional family structure. This is a strength for the questions addressed here, since such contexts are under-represented in the parenting literature, but it limits generalizability to urban and other cultural settings.

### Conclusion

This study examined associations between perceived parental attitudes and empathy among adolescents in two secondary schools in a rural province of Türkiye. Authoritarian parenting was negatively associated with empathy, both bivariately and after adjustment for demographic characteristics and the remaining parenting dimensions, and this was the most consistent finding across analyses. Protective/demanding parenting showed a small positive association that emerged more clearly under adjustment. Democratic parenting was not associated with empathy in this sample—a null that, given the study’s power and the adequate reliability of the measure, is better read as informative than indeterminate.

Two structural features of the data qualify these conclusions. The Democratic and Authoritarian subscales were moderately positively correlated, suggesting that warmth and control may not function as opposing dimensions in this context and raising questions about the cross-cultural portability of classical parenting typologies. And because the two participating schools were single-sex, sex and school context cannot be separated, so the observed sex difference—although consistent with a well-replicated literature—cannot be attributed to sex within this design.

The findings are best understood as identifying modifiable correlates of adolescent empathy within a specific sociocultural setting. They support attention to controlling parenting practices rather than to family structure, and they indicate that the applicability of Western parenting constructs in traditional family systems warrants direct examination rather than assumption. Longitudinal and multi-informant designs, together with measures distinguishing behavioral from psychological control, would be required to establish directionality and to test the interpretations offered here.

## Data Availability

The raw data supporting the conclusions of this article will be made available by the authors, without undue reservation.
