# Supplementary material for: Parental attitudes and adolescent empathy in a rural sociocultural context in Türkiye
Source: Front Psychol. 2026 Oct 22;17:1944408. doi: 10.3389/fpsyg.2026.1944408 (PMC13735541; doi:10.3389/fpsyg.2026.1944408)
Supplement: Supplementary file 1 [file Table_1.docx]

**Supplementary Material**

**Supplementary Table S1**

*PAS Total scores by students’ sociodemographic and academic characteristics*

| Characteristic | *n* (%) | PAS Total *M* (*SD*) | Test statistic |
| --- | --- | --- | --- |
| *Sex* |  |  |  |
| Female | 341 (74.5) | 116.76 (14.04) | *t*(456) = −1.41; *p* = .159; *d* = −0.15 |
| Male | 117 (25.5) | 118.95 (15.80) |  |
| *Age (years)* |  |  |  |
| 14 | 102 (22.3) | 115.46 (12.53) | *F*(4, 453) = 1.49; *p* = .204; *η*^2^ = .013 |
| 15 | 94 (20.5) | 118.12 (13.89) |  |
| 16 | 127 (27.7) | 117.80 (15.12) |  |
| 17 | 98 (21.4) | 119.22 (15.06) |  |
| 18 | 37 (8.1) | 113.81 (17.13) |  |
| *Grade level* |  |  |  |
| 9 | 139 (30.3) | 116.71 (13.26) | *F*(3, 454) = 0.42; *p* = .743; *η*^2^ = .003 |
| 10 | 55 (12.0) | 116.69 (17.40) |  |
| 11 | 195 (42.6) | 118.21 (15.06) |  |
| 12 | 69 (15.1) | 116.59 (13.05) |  |
| *Grade point average (n = 457)* |  |  |  |
| 85–100 | 62 (13.6) | 118.08 (15.48) | *F*(4, 452) = 0.40; *p* = .808; *η*^2^ = .004 |
| 70–84 | 191 (41.8) | 116.37 (13.25) |  |
| 60–69 | 107 (23.4) | 117.95 (16.29) |  |
| 50–59 | 77 (16.8) | 118.29 (14.49) |  |
| 0–49 | 20 (4.4) | 117.00 (14.08) |  |

*Note. N* = 458 unless otherwise indicated. PAS = Parental Attitude Scale. The PAS Total score is the arithmetic sum of the Democratic, Authoritarian and Protective/Demanding subscales and was therefore not entered into the regression models. Subscale scores for these grouping variables are reported in Table 1 of the main text. Two-group comparisons used independent-samples *t* tests with Cohen’s *d*; comparisons across three or more categories used one-way ANOVA with η². No comparison in this table remained significant after Benjamini–Hochberg correction. *p* values are two-tailed.

**Supplementary Table S2**

*PAS Total scores by family characteristics*

| Characteristic | *n* (%) | PAS Total *M* (*SD*) | Test statistic |
| --- | --- | --- | --- |
| *Mother's education* |  |  |  |
| Illiterate | 67 (14.6) | 114.54 (15.40) | *F*(4, 453) = 1.25; *p* = .288; *η*^2^ = .011 |
| Primary school | 243 (53.1) | 117.21 (14.84) |  |
| Secondary school | 96 (21.0) | 118.19 (11.60) |  |
| High school | 48 (10.5) | 120.31 (16.91) |  |
| University | 4 (0.9) | 114.50 (7.55) |  |
| *Father's education* |  |  |  |
| Illiterate | 14 (3.1) | 117.79 (17.23) | *F*(4, 453) = 0.69; *p* = .601; *η*^2^ = .006 |
| Primary school | 142 (31.0) | 118.49 (14.84) |  |
| Secondary school | 169 (36.9) | 116.02 (12.77) |  |
| High school | 110 (24.0) | 118.05 (16.63) |  |
| University | 23 (5.0) | 115.96 (12.37) |  |
| *Number of siblings* |  |  |  |
| 1–3 | 206 (45.0) | 119.22 (15.56) | *F*(2, 455) = 4.35; *p* = .014; *η*^2^ = .019 |
| 4–5 | 196 (42.7) | 116.50 (13.55) |  |
| 6 or more | 56 (12.2) | 113.30 (12.93) |  |
| *Parents' living arrangement* |  |  |  |
| Living together | 416 (90.8) | 117.37 (14.58) | *F*(2, 455) = 1.49; *p* = .227; *η*^2^ = .006 |
| Living separately | 21 (4.6) | 120.71 (13.00) |  |
| Parent deceased | 21 (4.6) | 113.00 (14.53) |  |
| *Family income* |  |  |  |
| Income below expenses | 158 (34.5) | 118.10 (17.42) | *F*(2, 455) = 0.34; *p* = .710; *η*^2^ = .002 |
| Income equal to expenses | 228 (49.8) | 116.92 (13.65) |  |
| Income above expenses | 72 (15.7) | 116.92 (9.43) |  |

*Note. N* = 458. PAS = Parental Attitude Scale. The PAS Total score is the arithmetic sum of the three subscales and was therefore not entered into the regression models. Subscale scores for these grouping variables are reported in Table 2 of the main text. Comparisons used one-way ANOVA with η². The only significant Tukey contrast was for number of siblings (1–3 > 6 or more, *p* = .017); no comparison in this table remained significant after Benjamini–Hochberg correction. *p* values are two-tailed.

**Supplementary Table S3**

*Hierarchical regression predicting adolescent empathy: two-subscale specification (sensitivity analysis)*

| Predictor | *B* | *SE* | 95% CI [LL, UL] | *t* | *p* | β | VIF |
| --- | --- | --- | --- | --- | --- | --- | --- |
| Intercept | 42.716 | 5.495 | [31.918, 53.514] | 7.77 | < .001 |  |  |
| Sex | 4.945 | 0.832 | [3.310, 6.580] | 5.95 | < .001 | .272 | 1.08 |
| Age | 0.614 | 0.290 | [0.044, 1.184] | 2.12 | .035 | .097 | 1.08 |
| Number of siblings | −0.716 | 0.234 | [−1.176, −0.255] | −3.05 | .002 | −.136 | 1.02 |
| PAS Authoritarian | −0.278 | 0.067 | [−0.410, −0.146] | −4.13 | < .001 | −.184 | 1.03 |
| PAS Protective/Demanding | 0.129 | 0.047 | [0.037, 0.221] | 2.75 | .006 | .124 | 1.05 |

*Note. N* = 458. PAS = Parental Attitude Scale; CI = confidence interval; LL = lower limit; UL = upper limit; *B* = unstandardised regression coefficient; β = standardised regression coefficient; VIF = variance inflation factor. Sex was coded 1 = female, 0 = male. Model fit: *R*² = .124, adjusted *R*² = .114, *F*(5, 452) = 12.85, *p* < .001.

This model is reported for transparency only. It reproduces the specification used in the originally submitted manuscript, in which the Democratic subscale was omitted. The specification reported in Table 4 of the main text, which enters all three subscales simultaneously, supersedes it. The two models yield the same substantive conclusions: authoritarian parenting is a significant negative predictor and protective/demanding parenting a significant positive predictor in both, with coefficients of comparable magnitude (β = −.184 vs. −.170, and .124 vs. .131, respectively).
